# Microbial dysbiosis and its diagnostic potential in androgenetic alopecia: insights from multi-kingdom sequencing and machine learning

**DOI:** 10.1128/msystems.00548-25

**Published:** 2025-05-28

**Authors:** Xiaochen Wang, Fengjuan Li, Yangyang Sun, Fan Meng, Yaolin Song, Xiaoquan Su

**Affiliations:** 1 College of Computer Science & Technology, Qingdao University 662302, Qingdao, Shandong, China; 2 Department of Dermatology, The Affiliated Hospital of Qingdao University 235960, Qingdao, Shandong, China; 3 Department of Pathology, The Affiliated Hospital of Qingdao University 235960, Qingdao, Shandong, China; Drexel University, Philadelphia, Pennsylvania, USA

**Keywords:** microbiome, scalp, skin, androgenetic alopecia, bioinformatics

## Abstract

Androgenetic alopecia (AGA), the most common form of hair loss, has been linked to dysbiosis of the scalp microbiome. In this study, we collected microbiome samples from the frontal baldness and occipital regions of patients with varying stages of AGA and conducted a comprehensive analysis of bacterial and fungal communities using 16S rRNA and ITS1 sequencing. Our results revealed that although the scalp microbiome dynamics in healthy subjects correlated strongly with chronological age, this trend was disrupted in AGA patients due to severe microbial imbalances, emphasizing the significant impact of AGA on the scalp microecology. Notably, microbial dysbiosis was not confined to the localized areas of hair loss but extended across the entire scalp. Moreover, the degree of dysbiosis was consistent with the severity of AGA. Leveraging multi-kingdom microbial features and machine learning, we developed a microbial index of scalp health (MiSCH), which effectively detects AGA and stratifies its severity. More importantly, MiSCH was able to identify high-risk individuals, those with significantly disrupted microbiome structures but no overt AGA phenotypic characteristics, thereby offering opportunities for early diagnosis, risk assessment, and personalized treatment of AGA.

**IMPORTANCE:**

By analyzing the bacteria and fungi on the scalp, this study shows how androgenetic alopecia (AGA) disrupts the balance of microbes not just in the hair loss areas, but across the entire scalp. Thus, we introduce the microbial index of scalp health (MiSCH), which leverages microbiome data for the early detection and severity prediction of AGA. This method is especially valuable for identifying people at risk of developing more severe hair loss, even before visible symptoms appear. By combining microbiome analysis with machine learning, this research offers a potential breakthrough for early diagnosis and personalized treatments, changing how we approach hair loss and offering new hope for managing the condition more effectively.

## INTRODUCTION

Androgenic alopecia (AGA) is a prevalent condition among Asians, with a male prevalence of approximately 21.3% in China. Over 250 million individuals in the country experience hair loss. Both men and women are affected by AGA, albeit with distinct patterns of hair loss and varying prevalence rates (1, 2). Globally, the number of individuals with hair loss is substantial. Factors such as irregular lifestyles, work-related stress, and societal pressures have contributed to an increased incidence of hair loss, with cases becoming more prevalent among younger populations. Consequently, there has been a significant rise in demand for anti-hair-loss products and medical interventions. Although AGA does not directly impact physical health, it severely affects mental well-being and quality of life (3). Early diagnosis and treatment are crucial, as they can significantly slow the progression of hair loss and improve the patient’s quality of life.

Characterized by the progressive miniaturization of hair follicles, AGA typically begins during adolescence or early adulthood. Repeated hair cycles in AGA shorten the anagen (growth) phase and increase the number of follicles in the telogen (resting) phase. Clinically, this results in thinning and shortening of hair, particularly in the frontal and parietal regions, eventually leading to scalp baldness (4–6). Numerous studies have demonstrated the involvement of genetic predisposition and hormonal alterations in the pathogenesis of AGA (7–9); however, research on monozygotic twins suggests that external factors also play a significant role in its progression (10). Environmental factors such as perifollicular inflammation, lifestyle stress, anxiety, and poor dietary habits can exacerbate AGA symptoms (11–13). Additionally, factors like sebum production and the microbial composition of the scalp further influence scalp health and hair loss (14, 15).

The skin, the body’s largest organ, serves as the primary barrier to environmental exposure and is colonized by a diverse array of microorganisms (16). The skin microbiome plays a critical role in maintaining skin and hair health (17, 18). It supports physiological homeostasis and protects against pathogen invasion (19). Emerging evidence has elucidated the relationship between the scalp microbiome and various scalp disorders, highlighting the contribution of microbial dysbiosis to conditions such as scalp allergies and hair loss (20). For instance, increased colonization by *g__Propionibacterium* in oily skin disrupts the skin barrier, exacerbating allergic reactions (21). Moreover, *g__Propionibacterium*, *g__Staphylococcus*, and *g__Malassezia* have been implicated in the pathogenesis of AGA and dandruff (22). Fungi such as *g__Malassezia*, *g__Aspergillus*, and *g__Staphylococcus* also serve as biomarkers for seborrheic dermatitis (23). These findings underscore the potential of the microbiome as a tool for better assessing and predicting disease states. However, the intricate interactions between AGA and the scalp microbiome remain poorly understood, warranting further investigation.

**Fig 1:**
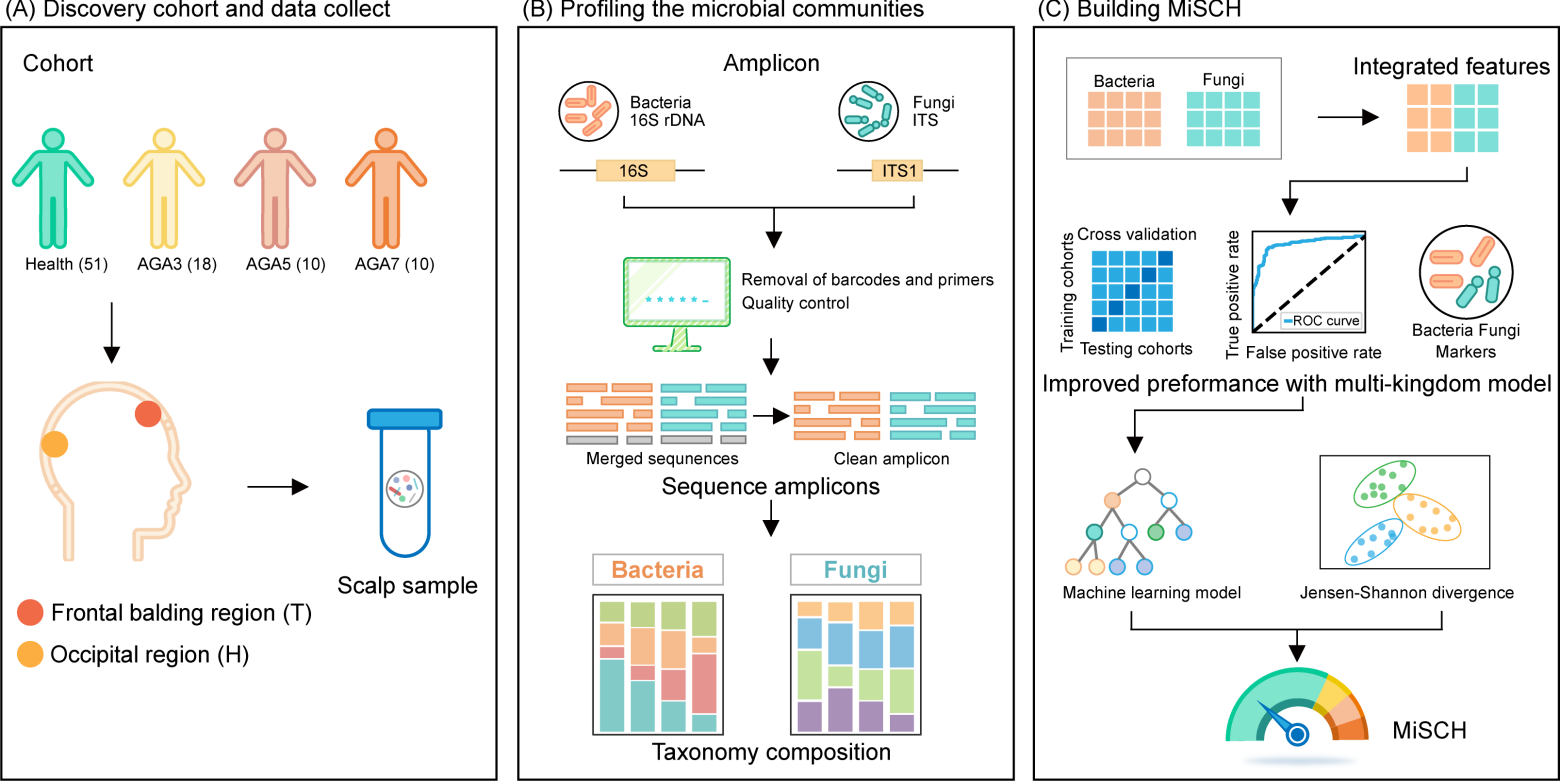
Overall diagram of experiment design and data analysis. (**A**) Cohort design and sample collection. (**B**) Microbial profiling and diversity analysis. (**C**) Machine learning and risk prediction by machine learning-based microbial index of scalp health (MiSCH).

In this study, we collected scalp samples from 89 adult individuals, including healthy controls and patients with varying degrees of AGA (stages 3, 5, and 7; Fig. 1A). To characterize the bacterial and fungal communities, we employed 16S rRNA and ITS1 amplicon sequencing, followed by bioinformatic analysis to explore the potential role of the scalp microbiome in AGA pathogenesis and the interrelationships within microbial communities. The results revealed a strong correlation between scalp microbiome and host age in healthy subjects, which was disrupted in AGA patients due to severe microbial dysbiosis (Fig. 1B). Based on such fundamental microbial patterns, we developed a machine learning model based on multi-kingdom microbiome features to assess and predict AGA states (Fig. 1C). This model addresses the limitations of traditional clinical approaches, which typically focus on diagnosing and staging AGA without the ability to predict disease progression.

## RESULTS

### Cohort design

This study involved the collection of scalp samples from 89 male participants residing in Shandong Province, China, including healthy individuals (*n* = 51) and patients with varying degrees of androgenic alopecia (stage 3 *n* = 18, stage 5 *n* = 10, and stage 7 *n* = 10; Table S1). Samples were collected from two regions of the scalp: the frontal bald area (T) and the occipital area (H) of each participant, resulting in a total of 178 specimens. The subjects’ ages ranged from 20 to 60 years. To better investigate scalp microbiota differences across age groups, we divided the cohort into 5-year intervals. In addition to the scalp samples, clinical and physiological data, including age and disease status, were recorded. The microbial community of bacteria and fungi of each sample was surveyed by 16S rRNA and ITS1 amplicon sequencing, respectively, and produced 356 sequence samples in total. A detailed description of the experimental design, cohort recruitment, and sequencing workflows is provided in Materials and Methods.

### Microbial age model reveals significant dysbiosis associated with AGA

As humans develop and age, the diversity and composition of the skin microbiome undergo significant changes. Previous work concluded that age is a key factor driving alterations in the skin microbiome (24) and also observed age-associated dynamics such as increases in *p__Actinobacteria* and *p__Proteobacteria* and a decline in *o__Lactobacillales* (25). Consistent with previous studies, the microbiome of healthy individuals in this study also exhibited significant changes across different age groups (Fig. S1 and S2). These findings indicate that the skin microbiome undergoes dynamic changes as the host matures (26, 27). However, it remains unclear whether androgenic alopecia onset or progression disrupts the healthy development of the scalp microbiome.

**Fig 2:**
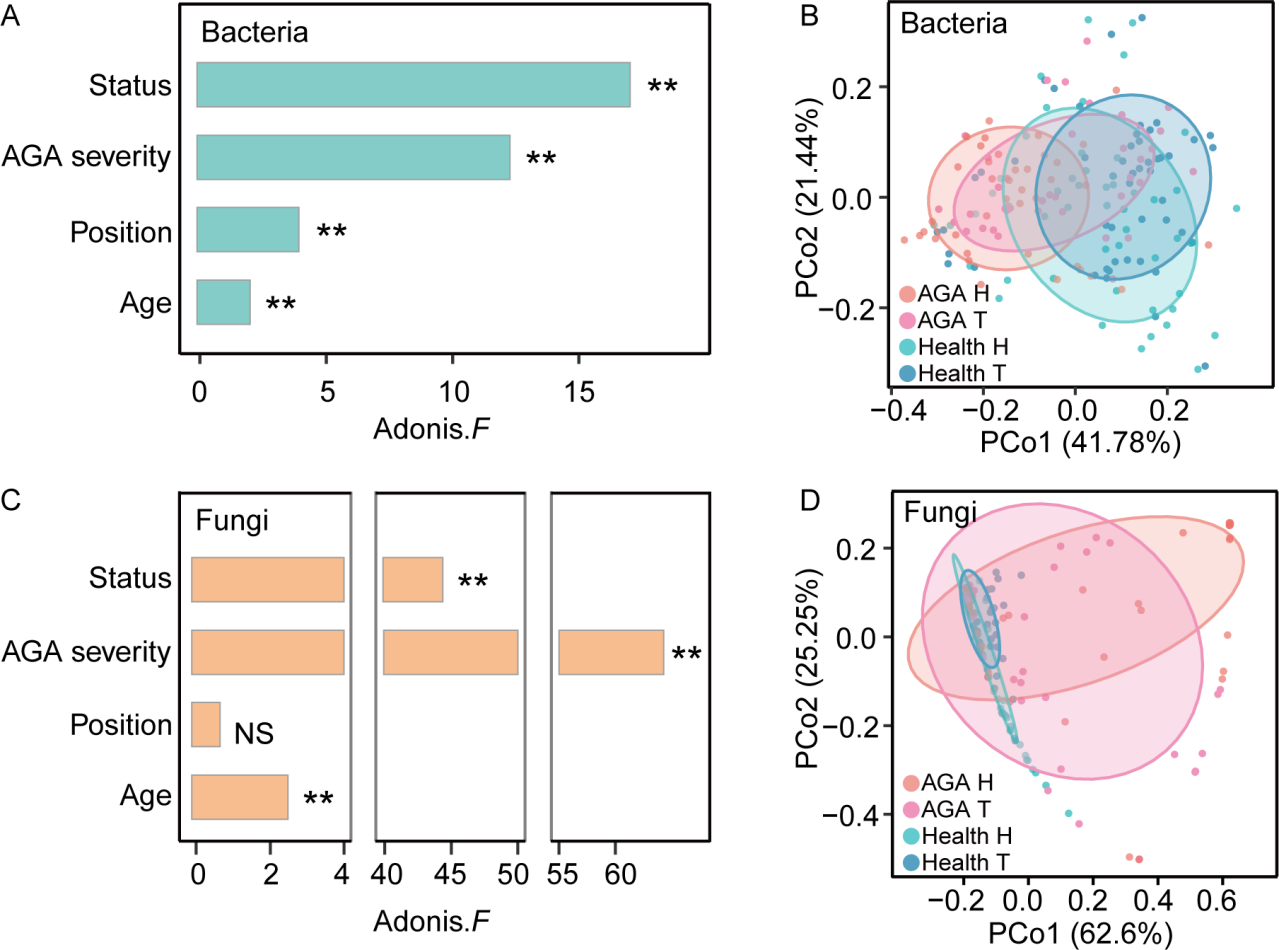
The effect size of various factors on scalp microbiota. (**A**) Effects of androgenic alopecia (AGA), AGA stages, sampling location, and host age on bacterial microbiota composition, where * indicates *P*-value < 0.05, ** indicates *P*-value < 0.01, and NS denotes not significant. (**B**) Beta-diversity analysis of bacterial microbiota at different sampling locations (frontal bald area and occipital area) in healthy individuals and AGA patients. (**C**) Effects of AGA, AGA stages, sampling location, and host age on fungal microbiota composition, where * indicates *P*-value < 0.05, ** indicates *P*-value < 0.01, and NS denotes not significant. (**D**) Beta diversity analysis of fungal microbiota at different sampling locations in healthy individuals and AGA patients.

In this work, we first assessed the influence of various factors on the scalp microbiome within the experimental cohort. Beta-diversity analysis showed that disease status had a significantly greater influence on the bacterial microbiome compared with either scalp region or host age (Fig. 2A). For fungi, disease status and severity were also the dominant factors affecting the microbiome, far surpassing the effects of other factors (Fig. 2C). These results suggest that the scalp microbial structure in AGA patients is markedly different from that of healthy controls, with fungal communities being more sensitive to changes associated with hair loss than bacterial communities. The variance analysis of alpha diversity on the Shannon index also exhibited, consistent with these observations, that identified disease status as the strongest effect factor of the scalp microbiome (Table S2).

We also compared microbial composition between the two scalp regions (T versus H) in both AGA patients and healthy individuals. The microbiome of the two regions in AGA patients exhibited similar compositions but differed significantly from those of healthy controls (Fig. 2B and C). This finding indicates that microbial imbalances in AGA patients are not restricted to the hair loss areas but extend across the entire scalp.

**Fig 3:**
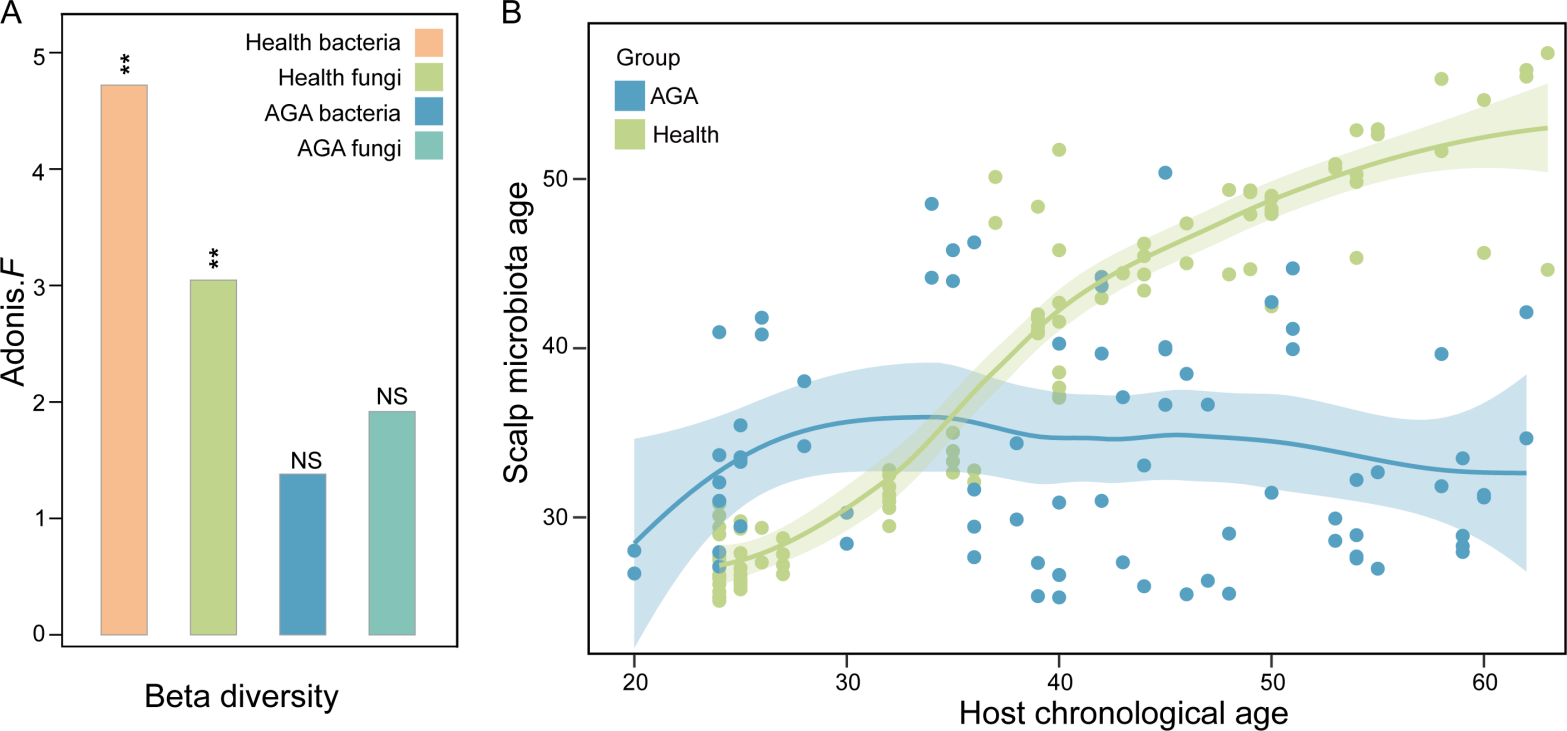
Scalp microbial age. (**A**) Beta-diversity of scalp bacterial and fungal microbiome in healthy subjects and AGA patients of different ages, where * indicates *P*-value < 0.05, ** indicates *P*-value < 0.01, and NS denotes not significant. (**B**) Scalp microbial age curves of healthy people and AGA patients.

Interestingly, although the microbial diversity in healthy individuals varied with age, the microbiome in AGA patients showed minimal age-related variation (Fig. 3A). Both bacterial and fungal community structures in AGA patients were less affected by age, highlighting the predominant influence of AGA on microbiome composition.

To further investigate the effects of AGA on the developmental trajectory of the scalp microbiome, we analyzed 102 scalp samples from healthy men aged 20–60 years. Using a robust random forest (RF) model (refer to Materials and Methods for details), we defined microbial age based on bacterial and fungal taxa. Cross-validation identified an optimal feature set, including five bacterial genera (*g__Paracoccus, g__Micrococcus, g__Rothia, g__Propionibacterium,* and *g__Acinetobacter*) and one fungal genus (*g__Cladosporium*), and these taxa were strongly correlated with the chronological age of the subjects (Table S3). This model revealed a strong correlation between microbial age and chronological age in healthy individuals, whereas the microbial age of AGA patients plateaued as the host’s age increased (Fig. 3B). The microbial age in AGA patients was significantly lower than that of healthy controls, indicating that AGA was associated with significant microbial dysbiosis, potentially contributed by disease progression.

### Alterations in scalp microbial composition associated with AGA

**Fig 4:**
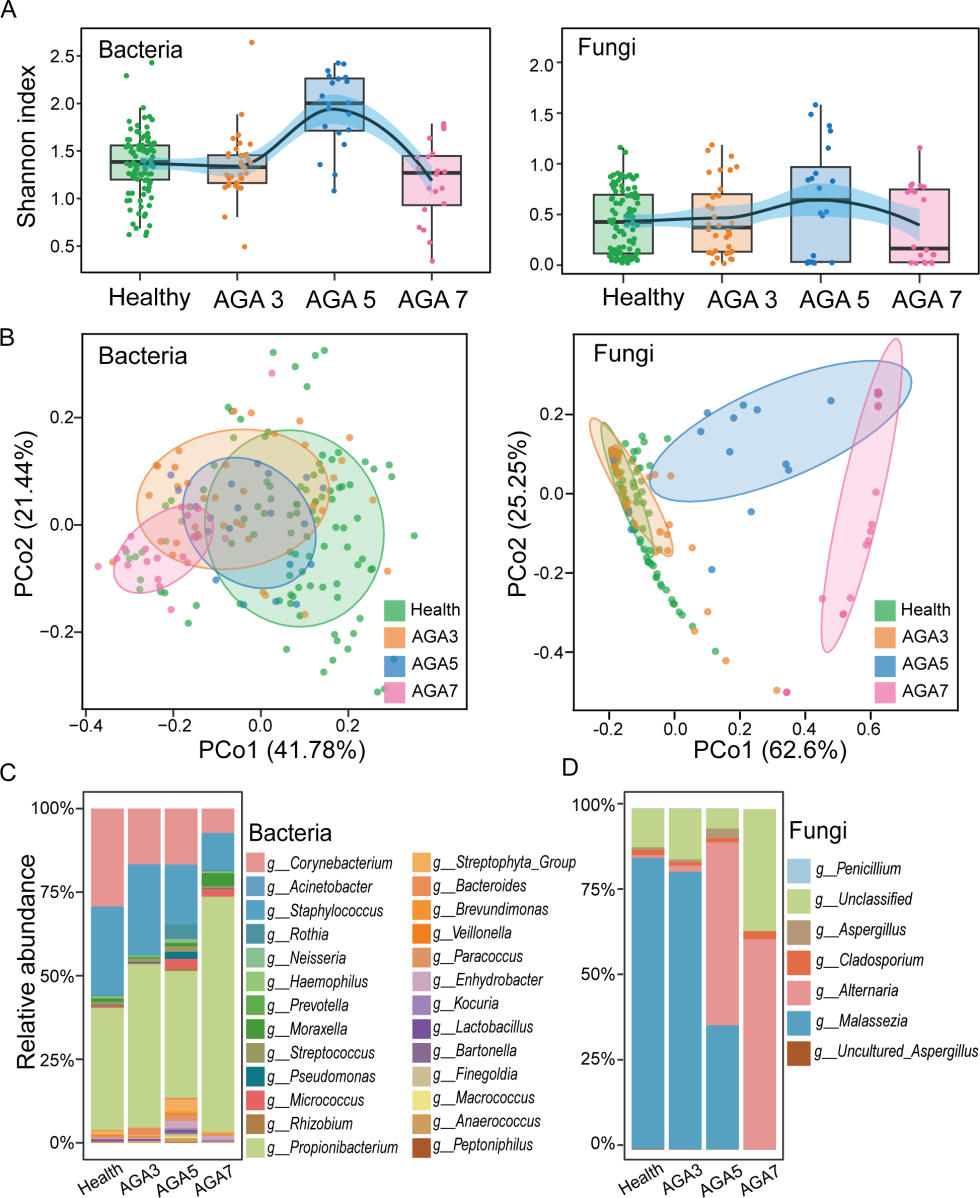
Variations in scalp microbial communities among healthy controls and patients with different stages of AGA. (**A**) Alpha-diversity analysis shows differences in bacterial and fungal communities between healthy individuals and patients at various stages of AGA, including changes in the Shannon index. (**B**) Principal coordinate analysis (PCoA) illustrates the clustering patterns of bacterial and fungal communities among healthy individuals and patients at various stages of AGA. (**C**) Composition of bacterial taxa at the genus level across healthy controls and AGA patients at different stages. (**D**) Composition of fungal taxa at the genus level across healthy controls and AGA patients at different stages.

The microbiome undergoes continuous changes during disease onset and progression (28–30), a pattern we also observed in the scalp microbiome of AGA patients. The Shannon index, which measures alpha-diversity, revealed that bacterial diversity in stage 3 AGA (AGA3) was comparable with that of healthy controls (Fig. 4A; Fig. S1). However, as hair loss severity increased, the Shannon index first rose, peaking at stage 5 (AGA5), before declining at stage 7 (AGA7). A similar trend was observed in fungal communities (Fig. 4A). We also measured the Chao1 index and Simpson index of the microbiome of healthy people and AGA patients at different stages and verified the above conclusions (Fig. S3). Beta-diversity analysis based on Jensen-Shannon divergence further demonstrated increasing deviations in microbial composition with advancing AGA severity. Healthy samples exhibited a closer microbial composition to AGA3 samples, whereas the distance between AGA3 and AGA5 samples, and AGA5 and AGA7 samples, grew progressively larger (Fig. 4B; Fig. S4).

Statistically significant differences were observed in the abundance of several bacterial genera (e.g., *g__Propionibacterium* and *g__Corynebacterium*) and fungal genera (e.g., *g__Malassezia*, *g__Alternaria*, and *g__Aspergillus*) between healthy individuals and those at various AGA stages. Many of these taxa displayed highly significant *P*-values that remained robust after false discovery rate (FDR) correction, highlighting their strong association with the onset and progression of AGA (Table S4). Further analysis revealed specific microbial shifts associated with AGA progression. The predominant genera in all groups were *g__Propionibacterium* and *g__Corynebacterium*, but dysbiosis was evident in AGA patients (Fig. 4C; Fig. S5). Specifically, *g__Propionibacterium* was enriched in AGA patients, likely driven by sebaceous gland hyperplasia, which provides lipids and fatty acids as substrates for *g__Propionibacterium*. The overgrowth of *g__Propionibacterium* may induce inflammatory responses, contributing to hair follicle miniaturization and exacerbating hair loss (14). Conversely, *g__Corynebacterium* abundance decreased with increasing AGA severity. In fungal communities, *g__Alternaria* remained at low abundance in healthy individuals and early AGA stages (AGA3), but its abundance increased significantly as hair loss progressed, surpassing 50% in AGA7 samples (Fig. 4D; Fig. S5). The lipophilic fungus *g__Malassezia* also declined in abundance as hair loss severity increased, whereas *g__Aspergillus* initially increased, peaking at AGA5, before decreasing.

These microbial dynamics highlight significant differences in the microbial composition between various stages of AGA, emphasizing the potential of the scalp microbiome as a biomarker for early AGA detection. Microbial shifts can effectively differentiate healthy individuals from patients with different stages of AGA, supporting the use of microbial profiling in AGA diagnosis and prognosis.

### A machine learning-based index for AGA severity assessment

Although AGA is typically diagnosed based on clinical symptoms and characteristic hair loss patterns, variability in diagnosis and the progressive nature of the disorder can make prognosis challenging. Traditional diagnostic methods are limited in predicting disease progression, especially for patients with mild or moderate AGA. To address this limitation, we introduced the microbial index of scalp health (MiSCH), which combines a multi-class classification model with microbial community distance metrics to quantify AGA severity and predict disease risk.

**Fig 5:**
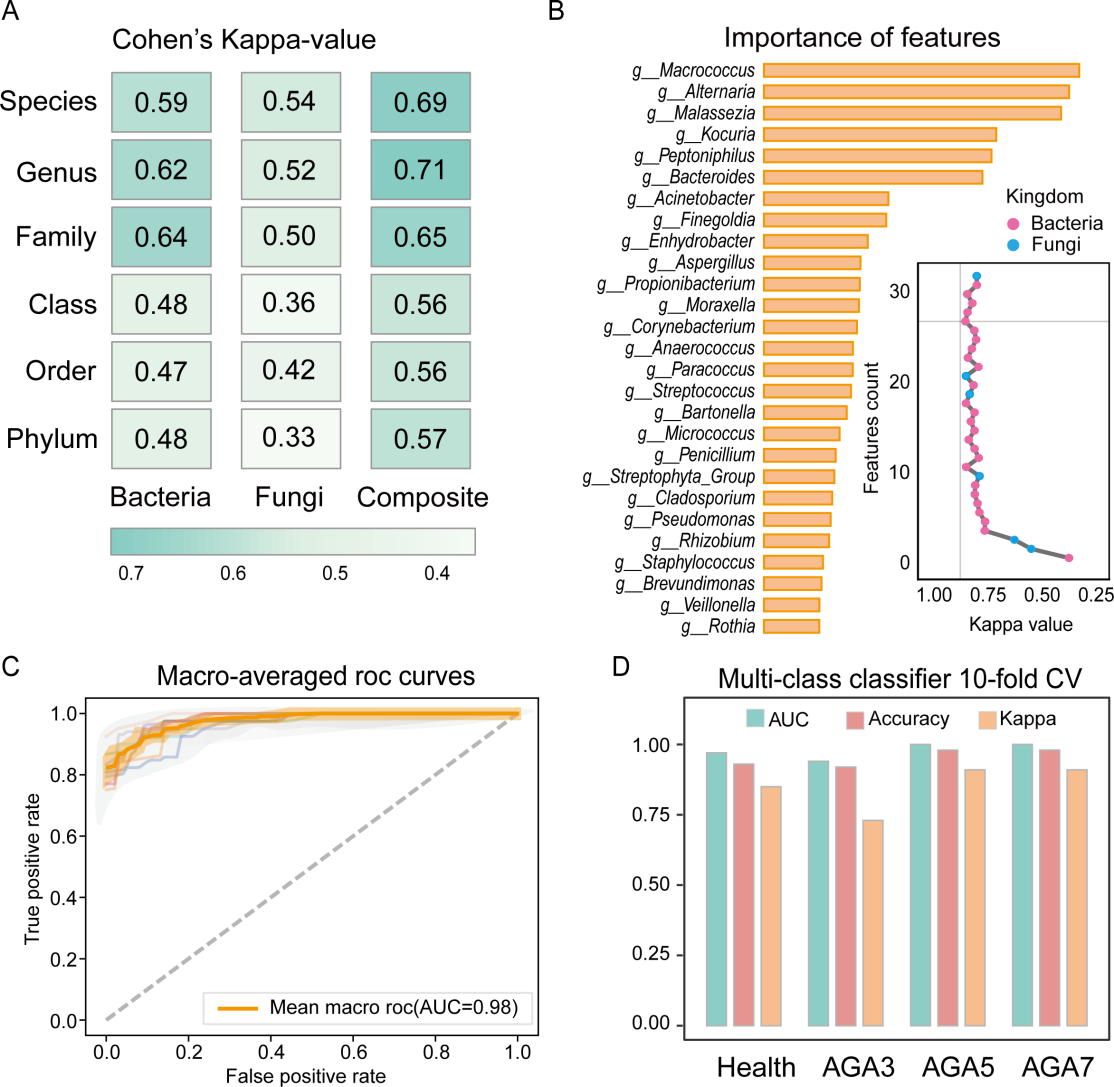
Construction and performance of the AGA multi-class classification model. (**A**) Model performance was evaluated across six taxonomic classification levels using bacterial, fungal, and combined bacterial and fungal features, respectively. (**B**) Feature importance ranking using a random forest model. The feature set was incrementally expanded according to the ranking results, with the top 22 bacterial and five fungal species identified as having the strongest multi-class classification ability. (**C**) Model performance was assessed over 10 iterations using different data splits, with the arithmetic mean of the one-vs-rest (OvR) area under the curve (AUC) values calculated for all classes in each test. (**D**) Average model performance across 10 test iterations, including metrics such as AUC, accuracy, and Cohen’s kappa, calculated using the OvR method.

First, we developed a random forest (RF) multi-class classification model using all 178 male scalp samples from healthy controls and AGA patients. To assess whether model performance was influenced by taxonomic annotations, we built classifiers at six taxonomic levels, ranging from kingdom to species, and evaluated performance using 10-fold cross-validation. We also evaluated classifiers based on bacterial features, fungal features, and combined bacterial and fungal features. The highest kappa value was achieved when bacterial and fungal data were integrated at the genus level (Fig. 5A).

To further optimize the model, we identified key biomarkers at the genus and species levels from the combined bacterial and fungal data sets. By selecting the top 27 most discriminative genera (22 bacterial and five fungal), we observed a peak in the kappa value (Fig. 5B). Additional taxa beyond these 27 did not significantly improve performance, suggesting these genera had the strongest discriminative ability and are optimal as AGA biomarkers. Notably, *g__Macrococcus* emerged as the most prominent biomarker, whereas fungal genera such as *g__Alternaria* and *g__Malassezia* also played critical roles in the model. It is interesting that the predictive power of multi-kingdom markers has also been validated by calculating the macro-averaged receiver operating characteristic (ROC) of multi-class classification models (Fig. 5C). Ultimately, we constructed a robust multi-class classification model that leveraged the combined bacterial and fungal microbiota (Fig. 5D).

**Fig 6:**
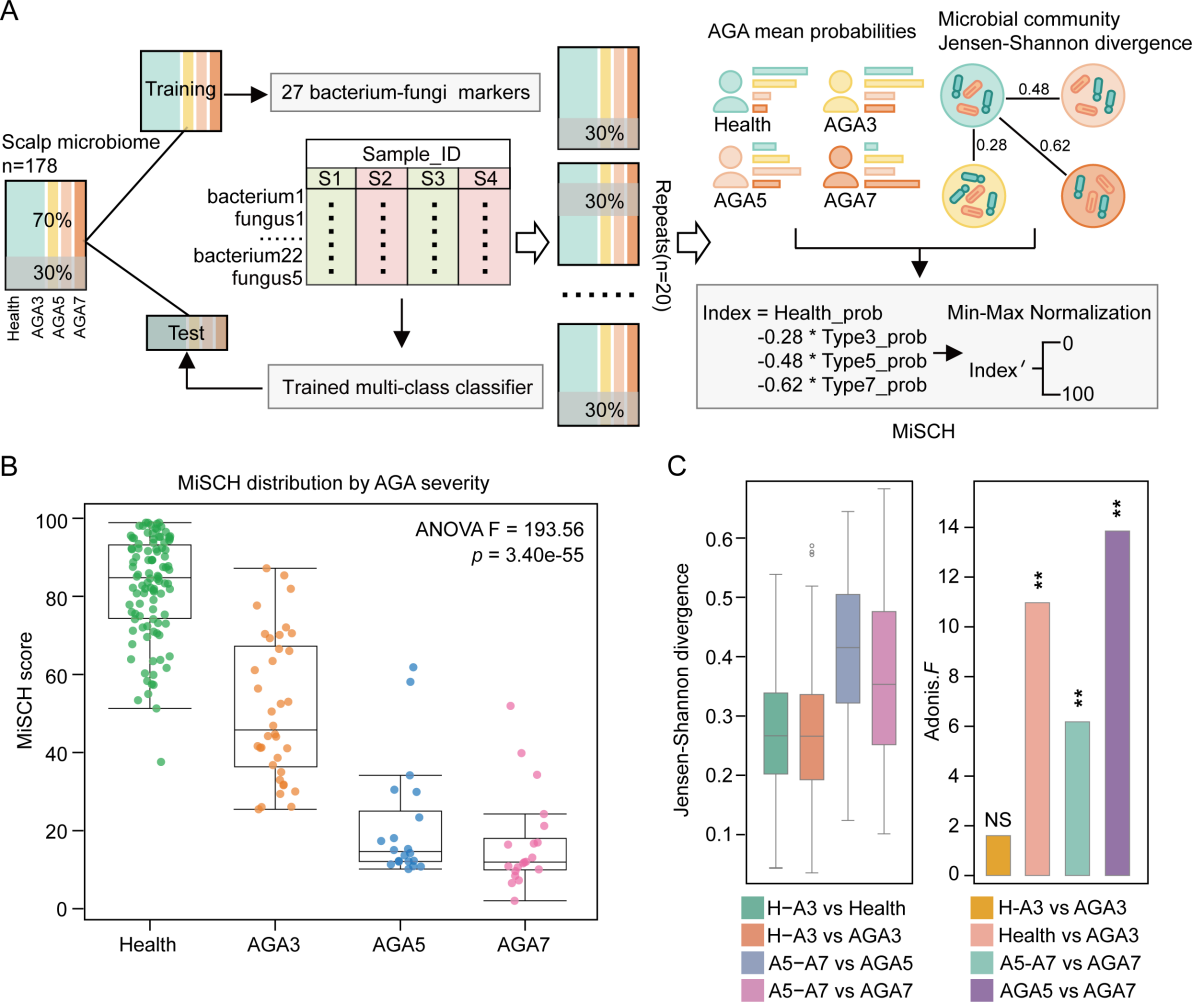
Construction of MiSCH. (**A**) Based on the multi-class classification prediction results and the microbial distances between the healthy microbiome and different types of AGA microbiome, an index score (MiSCH) between 0 and 100 was obtained. (**B**) MiSCH scores were strongly correlated with AGA hair loss symptoms. (**C**) The microbiome structure similarity of high-risk samples identified by MiSCH was more akin to that of malignant samples, where * indicates *P*-value < 0.05, ** indicates *P*-value < 0.01, and NS denotes not significant. H-A3 represents healthy phenotype samples misdiagnosed as AGA3 by MiSCH, and A5-A7 represent samples misdiagnosed as AGA7 from an AGA5 phenotype by MiSCH.

Based on the RF model and Jensen-Shannon divergence across microbiota at different stages of AGA (refer to Materials and Methods for details), we established a microbial index of scalp health (MiSCH; Fig. 6A), which quantitatively measures the AGA severity. MiSCH produces an index score ranging from 0 to 100, with higher scores indicating a healthier scalp microbiome and lower scores associated with more severe AGA symptoms. The scores were divided into four categories: healthy group (100 ≥ MiSCH > 75), mild hair loss (75 ≥ MiSCH > 25), moderate hair loss (25 ≥ MiSCH > 15), and severe hair loss (15 ≥ MiSCH). A strong correlation was observed between MiSCH scores and clinical AGA assessments, confirming the index’s accuracy in distinguishing among AGA severity levels (Fig. 6B; ANOVA, *F* = 193.56, *P*-value = 3.40 × 10^−55^).

### Prediction of AGA risk by MiSCH

Interestingly, when applied to detect scalp samples, MiSCH identified certain samples that exhibited microbiome characteristics indicative of more severe AGA, which we refer to as “high-risk” samples. Despite these samples showing mild AGA symptoms based on hair loss patterns, their microbiome structure resembled that of more advanced AGA cases. In the experimental cohort, MiSCH classified 27 healthy samples as AGA3 (designated as H–A3) and 10 AGA5 samples as AGA7 (designated as A5–A7).

The microbiota of the A5–A7 samples showed greater similarity to that of AGA7 samples, with a closer Jensen-Shannon divergence between these more severe AGA microbiota. Adonis analysis revealed that the *F* for the comparison between A5–A7 and AGA7 microbiota was smaller (Fig. 6C), further supporting this similarity. Additionally, no significant difference in microbiome structure was found between H–A3 and AGA3 samples (Fig. 6C), emphasizing that the microbiota of high-risk samples more closely aligned with that of advanced AGA cases.

Among all 19 inconsistently identified samples between MiSCH and microbiota beta-diversity pattern, MiSCH exhibited strong discrimination ability with a weighted F1 score of 0.89 (specificity = 0.90, sensitivity = 0.73). In summary, even in the absence of visible changes to a subject’s hair phenotype, the scalp microbiome is already showing signs of disruption, signaling an early shift toward a more pathological microbiome state. Thus, MiSCH can serve as a valuable tool for predicting the future trajectory of AGA, allowing for early intervention before clinical symptoms become apparent.

### Conclusion and discussion

This study provides compelling evidence for the role of the scalp microbiome in androgenetic alopecia (AGA) and proposes a novel microbiome-based index, MiSCH, for predicting AGA progression. By analyzing the microbial communities of the scalp in individuals with varying degrees of AGA, we have identified significant alterations in both bacterial and fungal compositions that correlate with disease severity. Our findings demonstrate that the scalp microbiome is not only disrupted in areas of active hair loss but is also altered across the entire scalp in AGA patients, with microbial dysbiosis serving as an early indicator of disease onset and progression.

Through detailed microbiome profiling, we observed that the disease status, rather than age or scalp region, had the most pronounced impact on both bacterial and fungal communities. The microbial structure in AGA patients exhibited a distinctive pattern compared with healthy controls, with certain genera, including *g__Propionibacterium* and *g__Corynebacterium*, showing notable shifts in abundance as the disease progressed. Importantly, we found that microbial diversity in AGA patients was less influenced by age, highlighting that the disease itself significantly alters the normal maturation process of the scalp microbiome.

Our approach incorporated both bacterial and fungal data, leading to the development of the Microbial Index of Scalp Health (MiSCH), a model that can accurately classify AGA stages and assess disease risk. By identifying key microbial biomarkers and constructing a robust multi-class classification model, we demonstrated that MiSCH provides a quantitative measure of AGA severity. The model showed excellent predictive power, with a strong correlation between microbiome-derived MiSCH scores and clinical evaluations of AGA severity. More importantly, MiSCH was able to identify “high-risk” patients, whose microbiome was already showing signs of severe AGA despite their clinical presentation indicating mild or no hair loss. This underscores the potential of microbiome-based tools to detect AGA in its earliest stages and predict its future progression.

The implications of this study are significant for both the early diagnosis and management of AGA. Traditional diagnostic approaches, which are primarily based on clinical appearance and patient history, are limited in predicting disease progression. The introduction of MiSCH provides a powerful alternative, enabling clinicians to identify at-risk individuals before visible symptoms of AGA become apparent. Furthermore, this microbiome-based model could be used to monitor treatment efficacy and guide personalized therapeutic interventions. For example, the microbial diversity (Fig. 4B; Fig. S4) shows only subtle differences between healthy individuals and those with stage 3 AGA, suggesting that stage 3 may represent a window for early diagnosis and intervention. Initiating treatment at this stage may potentially slow or halt disease progression and also enable restoration of the scalp microbiome to a healthier state with minimal intervention. This possibility warrants further validation through longitudinal or interventional studies.

Although our findings provide strong evidence for the role of the scalp microbiome in AGA, several questions remain. Future research should focus on the causal relationships between microbial dysbiosis and hair loss. It is still unclear whether the changes in the microbiome are a direct cause of AGA or a consequence of the disease. Additionally, although our study emphasizes the importance of specific microbial genera, the interactions within the microbiome as a whole, such as the relationship between bacteria and fungi, warrant further exploration. Understanding these interactions could enhance our ability to target the microbiome for therapeutic purposes.

In conclusion, this study highlights the significant role of the scalp microbiome in AGA and presents MiSCH as a promising tool for diagnosing and predicting the progression of this common condition. By leveraging microbiome data, clinicians can gain deeper insights into the pathophysiology of AGA, enabling more accurate risk assessments, earlier interventions, and personalized treatments. Moving forward, the integration of microbiome-based diagnostics in clinical practice has the potential to revolutionize the management of AGA and offer new avenues for therapeutic development.

## MATERIALS AND METHODS

### Experimental design and sample collection

A cohort consisting of 38 male patients with varying stages of AGA and 51 healthy male controls was recruited from Shandong, China. We selected subjects at AGA stages 3, 5, and 7 based on criteria including key disease progression stages, inter-group significance, and sample availability. According to the Hamilton-Norwood classification (31), these stages represent mild, moderate, and severe hair loss, respectively, with stage 3 marking the onset of visible hair thinning, and stages 5 and 7 reflecting advanced AGA with notable changes. This targeted selection enabled us to capture meaningful microbiota differences while avoiding over-fragmentation that would reduce model stability and usability. AGA diagnoses were made by expert dermatologists from the Affiliated Hospital of Qingdao University, based on the Hamilton-Norwood (31) classification. Exclusion criteria included individuals with skin diseases other than AGA and those who had used drugs to promote hair regeneration or slow the progression of hair loss. Subjects were instructed not to wash their hair or wipe their scalp with a wet towel from the evening before sample collection until the day of collection. Additionally, they were asked to avoid making major changes to their daily routines (e.g., strenuous exercise). Each participant’s scalp was examined, and samples were collected from both the frontal hair loss area and the posterior scalp. The scalp samples were sent to the hospital within 24 h of collection and stored at −80°C until further processing. A total of 178 scalp skin specimens from 89 subjects were included for subsequent data analysis.

### DNA extraction, sequencing, and data processing

A total of 178 scalp specimens were collected for DNA extraction, 16S rRNA amplicon sequencing, and ITS1 sequencing. Genomic DNA was extracted from the skin specimens using the TIANamp Swab DNA Kit (TIANGEN, Inc., China), following the manufacturer’s instructions. Universal primers were employed to amplify the bacterial 16S rRNA gene (v1-v3 region) and the fungal ITS1 region. The resulting PCR products were automatically purified using the Agencourt AMPure XP (Beckman Coulter, Inc., USA) nucleic acid purification kit.

The purified PCR products were then constructed into libraries using the NEB Next Ultra II DNA Library Prep Kit (New England Biolabs, Inc., USA). 16S and ITS1 sequencing were performed on the Illumina MiSeq and NovaSeq 6000 platforms (Illumina, Inc., USA), respectively, using paired-end sequencing strategies (PE300 and PE250). A total of 356 sequence samples were produced (178 16S and 178 ITS1).

RAW sequencing data were processed using QIIME (32) (v1.8.0) software, which sorted the sequences based on barcode sequences. The data were then filtered and spliced using the PEAR (33) (v0.9.6) software, removing sequences with scores lower than 20, ambiguous bases, or primer mismatches. The minimum overlap for splicing was set to 10 bp, and the mismatch rate was set to 0.1.

For the 16S rRNA gene data, sequences shorter than 230 bp were removed after splicing, and chimeric sequences were identified and discarded using the UCHIME (34) method, based on the Gold Database. For ITS1 sequences, sequences shorter than 120 bp were removed post-splicing, and chimeric sequences were eliminated using the UCHIME method, based on the Unite database. OTU clustering and species annotation were performed using the Parallel-Meta Suite (35) (v3.7.3). The representative OTU sequences for 16S rRNA were compared against the Greengenes database (v13.8), whereas the ITS1 OTUs were compared against the Unite database (v8.2).

### Comprehensive analysis of scalp microbiota

Alpha-diversity was assessed using the Shannon index, Simpson index, and Chao1 index, based on genus-level profiles (36). We performed multiple linear regression analysis and analysis of variance for each categorical variable and exported statistical significance (*P-*value) to quantify the relative contribution of each factor to the alpha-diversity of the scalp microbiome. The statistical significance between groups was evaluated using the Wilcoxon rank-sum test, and the Spearman correlation coefficient was used for correlation analysis. To assess the dissimilarity between samples, the Jensen-Shannon divergence (37) was calculated. Additionally, the Adonis analysis (also known as permutation multivariate analysis of variance [PERMANOVA]) was performed to evaluate the statistical significance of beta diversity and the effect size of host variables. A *P*-value threshold of 0.01 was set for statistical significance. In PCoA analysis, we draw circles or ellipses with 85% confidence intervals to show the range of variation and distribution trends within the group. All diversity and statistical analysis procedures were conducted using Parallel-Meta Suite (v3.7.3).

### Construction of the microbial age model

To investigate the microbial age of the scalp microbiome, we analyzed a data set of healthy individuals aged 20–60 years. A regression model was constructed using genus-level bacterial and fungal taxa along with the host’s chronological age, implemented via the RandomForestRegressor module (v3.11.8) in Python. Microbial age was then estimated using this model. To identify key microbial features, we calculated Gini importance scores via the feature_importances_ attribute. For robustness, 90% of the samples were randomly selected for each iteration of feature ranking, and this process was repeated 10 times. The final feature importance ranking was obtained by averaging the scores across these iterations. Based on this ranking, features were incrementally added to the model, and 10-fold cross-validation was used to evaluate performance at each step. This iterative process led to the identification of an optimal feature set comprising both bacterial and fungal genera. Using this refined feature set, we constructed a robust random forest model capable of accurately predicting microbial age based on microbiome composition.

### Predictive modeling of the onset of AGA

A multi-class classification model was implemented using Python 3.11.8, utilizing standard library implementations: pandas (v2.2.1), numpy (v1.26.4), scikit-learn (v1.4.1), and matplotlib (v3.8.3). Random forest was employed as the classification model with default parameters to diagnose varying stages of AGA severity based on the classification of scalp bacterial, fungal, and combined bacterial-fungal microbiota. Stratified K-fold cross-validation was performed with 10-fold, and the Cohen’s kappa score was used to evaluate the performance of the model at different taxonomic levels for each kingdom and species classification.

The genus level of the bacterial-fungal microbiota demonstrated the best classification accuracy. At the genus level, the feature_importances_ function was used to select the 27 bacterial and fungal species with the highest classification ability. For each phenotype, the samples were randomly divided into training and test sets, with the training set comprising 70% of the total samples (178 samples in total). The samples from the H site and T site of the same individual were assigned exclusively to either the training set or the test set. For hyperparameter tuning, we first conducted a broad search of 200 parameter combinations using RandomizedSearchCV, then refined our results with an exhaustive GridSearchCV over 648 configurations within a narrower range (Table S5). This data partitioning process was repeated 10 times, and the average performance across the 10 test sets was used as the final model performance to predict the severity of AGA.

### Construction of MiSCH

The comprehensive microbial distance was calculated by integrating normalized relative abundance tables of bacterial and fungal taxa into a unified data set. Using this data set, the Jensen-Shannon divergence was employed to measure the differences in microbial composition between samples with varying stages of AGA.

We analyzed data from 178 healthy individuals and AGA patients, using genus-level information to construct a multi-class classification model based on the optimal multi-kingdom microbiota. The data set was randomly divided into training (70%) and testing (30%) sets, and we assigned a unique number to each subject to ensure that samples from different scalp regions of the same subject were exclusively assigned to the training or testing set. The trained model generated probability distributions for each AGA severity category, producing a multi-category probability array for each sample. This process was repeated 20 times, and the average probabilities were used as the final predicted probabilities for each category.

Unlike models that directly output a symptom label, our health index integrates microbial distances between the healthy microbiome and the comprehensive microbiome associated with different AGA severities. We calculated the Jensen-Shannon divergence between each healthy sample and each sample within a given AGA stage group. The average of all pairwise distances was then used to represent the overall microbial distance between that AGA group and the healthy control group. This distance is used as a weight, combined with the probabilities of each category label derived from the multi-class classification model, to produce an index score ranging from 0 to 100. This prediction score reflects the association between microbiome composition and AGA severity.

To ensure MiSCH’s effectiveness in distinguishing among AGA severity levels, we defined score intervals corresponding to four categories: healthy group (100 ≥ MiSCH > 75), mild hair loss (75 ≥ MiSCH > 25), moderate hair loss (25 ≥ MiSCH > 15), and severe hair loss (15 ≥ MiSCH). These thresholds ensure a high degree of accuracy in differentiating between health and various stages of AGA.

In evaluating overall scalp health, we prioritized the lower of the two MiSCH scores derived from the frontal and occipital regions. A lower MiSCH score indicates more severe microbial dysbiosis and is therefore considered a more accurate reflection of disease severity. This approach allows for a more conservative and clinically relevant assessment of AGA risk.

## Data Availability

The sequence data in this study have been submitted to the Sequence Read Archive (https://www.ncbi.nlm.nih.gov/sra) and can be accessed through the BioProject number PRJNA1223116. The Strengthening the Organizing and Reporting of Microbiome Studies (STORMS) checklist is available at https://doi.org/10.5281/zenodo.15208862. Source codes and scripts for data analysis are available at the GitHub repository at https://github.com/qdu-bioinfo/MiSCH.

## SUPPLEMENTAL MATERIAL

Supplemental information msystems.00548-25-s0001.docx Fig. S1 to S5; Tables S1 to S5.

ASM does not own the copyrights to Supplemental Material that may be linked to, or accessed through, an article. The authors have granted ASM a non-exclusive, world-wide license to publish the Supplemental Material files. Please contact the corresponding author directly for reuse.
