# Supplementary material for: Microbial dysbiosis and its diagnostic potential in androgenetic alopecia: insights from multi-kingdom sequencing and machine learning
Source: mSystems. 2025 May 28;10(6):e00548-25. doi: 10.1128/msystems.00548-25 (PMC12172500; doi:10.1128/msystems.00548-25)
Supplement: Supplemental information — Fig. S1 to S5; Tables S1 to S5. [file msystems.00548-25-s0001.docx]

**Supplementary information**


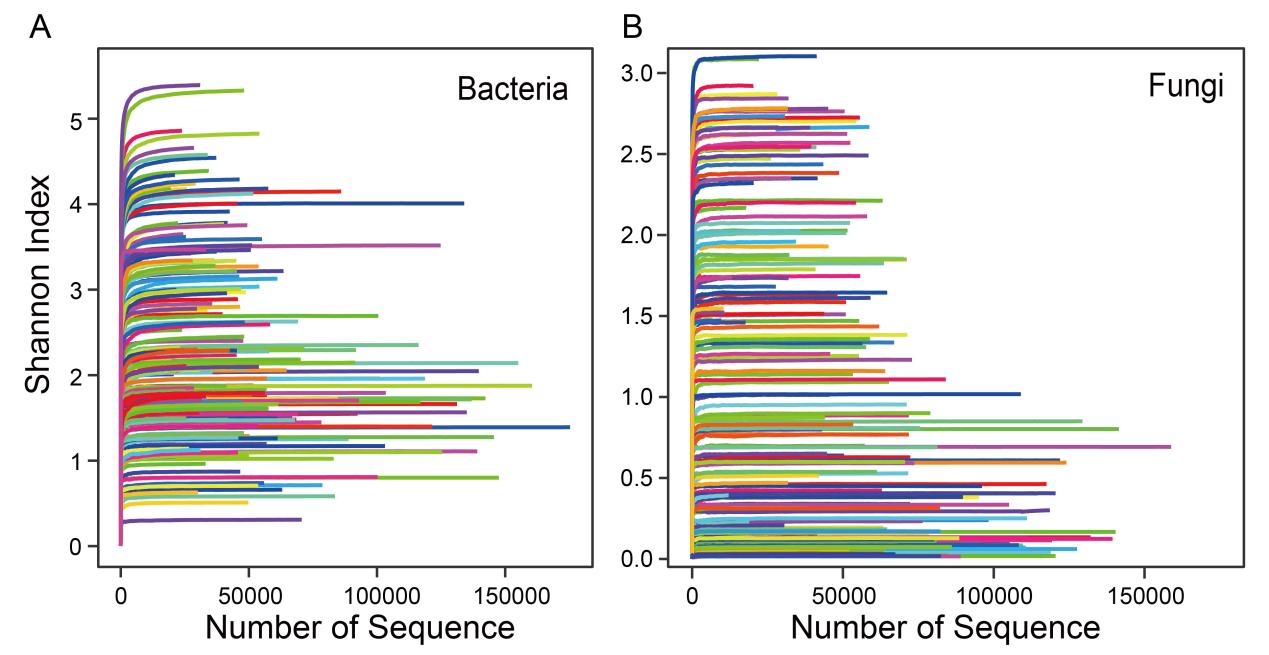


**Fig. S1. Rarefaction curves of Shannon index using genus-level (A) bacterial and (B) fungal features from the experimental cohort.**


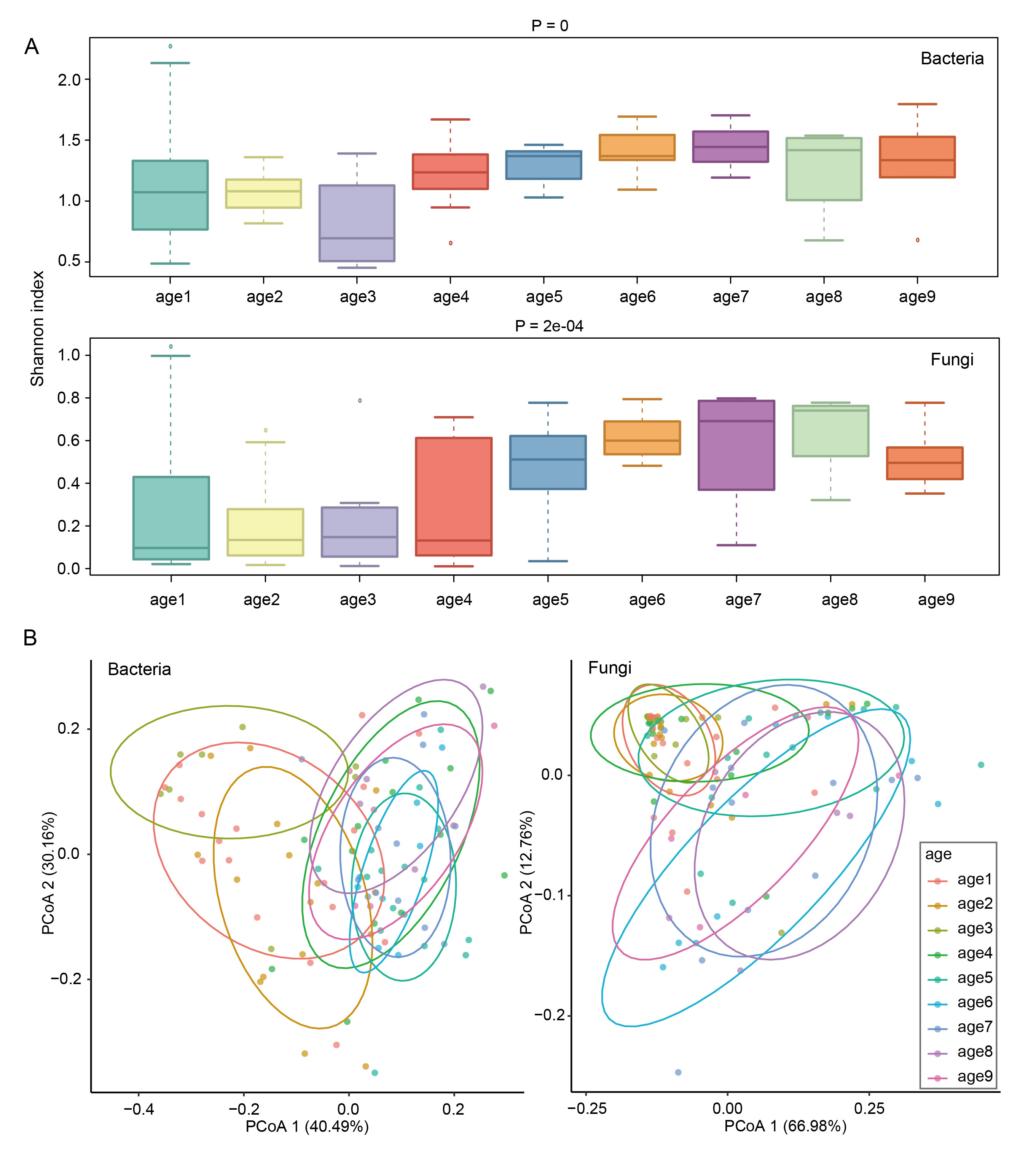


**Fig. S2. Changes in the fragrance index (A) and PCoA analysis (B) of fungi and bacteria in healthy people of different age groups.**


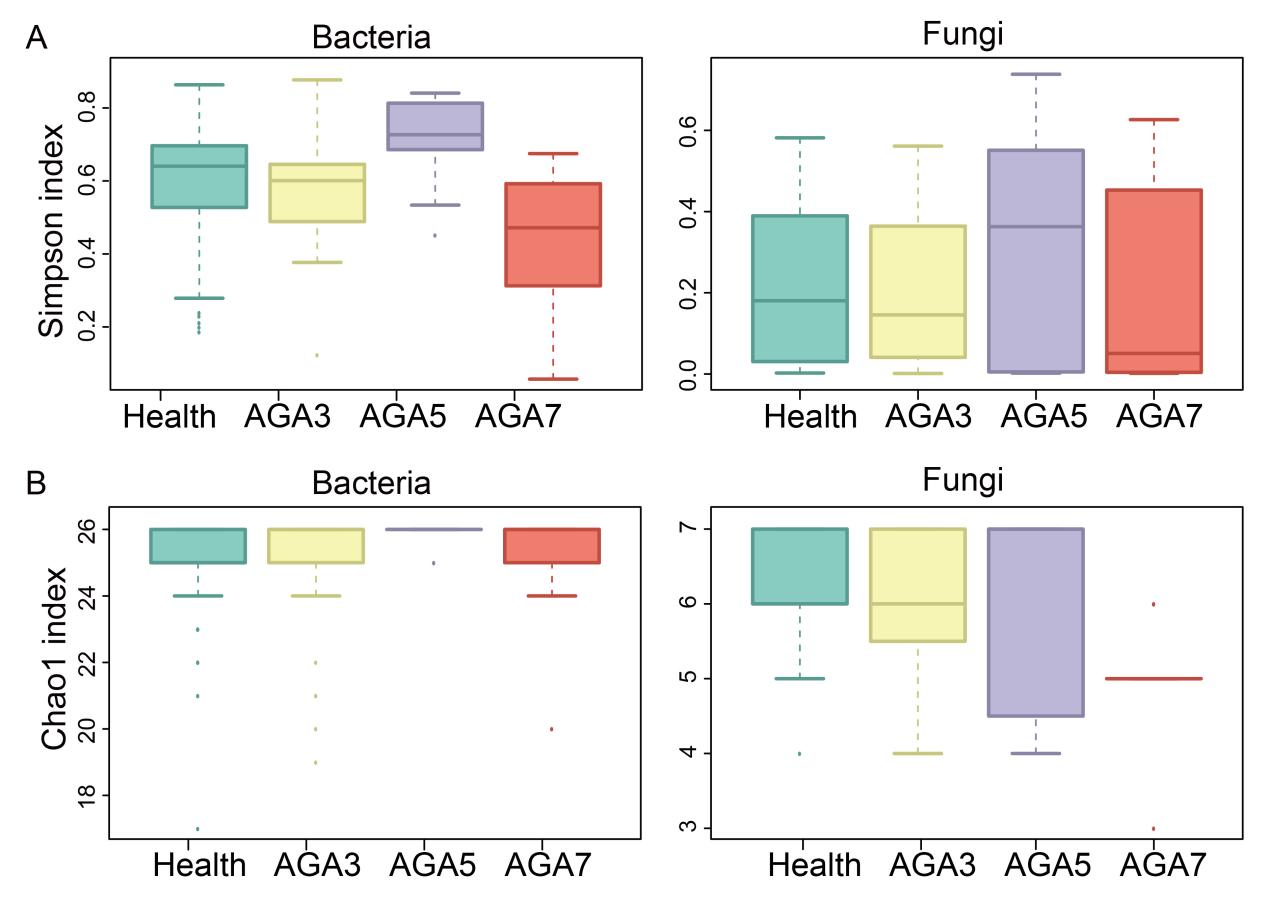


**Fig. S3. Changes in (A) Simpson index and (B) Chao1 index of bacteria and fungi in healthy populations of AGA patients at different stages.** The microbial alpha diversity at AGA3 was similar to that of the healthy control group, and the values increased with increasing severity of hair loss, reaching a peak at AGA5 and then decreasing at AGA7. This conclusion was also verified in terms of the Chao1 index and Simpson index.


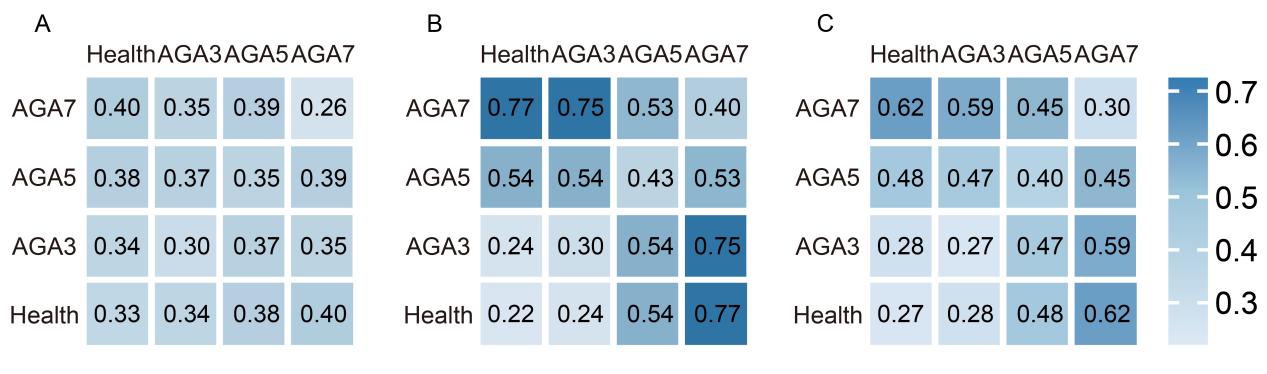


**Fig. S4. Microbial distances between healthy people and patients with different degrees of AGA, calculated using the Jensen-Shannon divergence.** (A) Distances based on bacterial genus-level features. (B) Distances based on fungi genus-level features. (C) Distances based on composite genus-level features of bacteria and fungi features


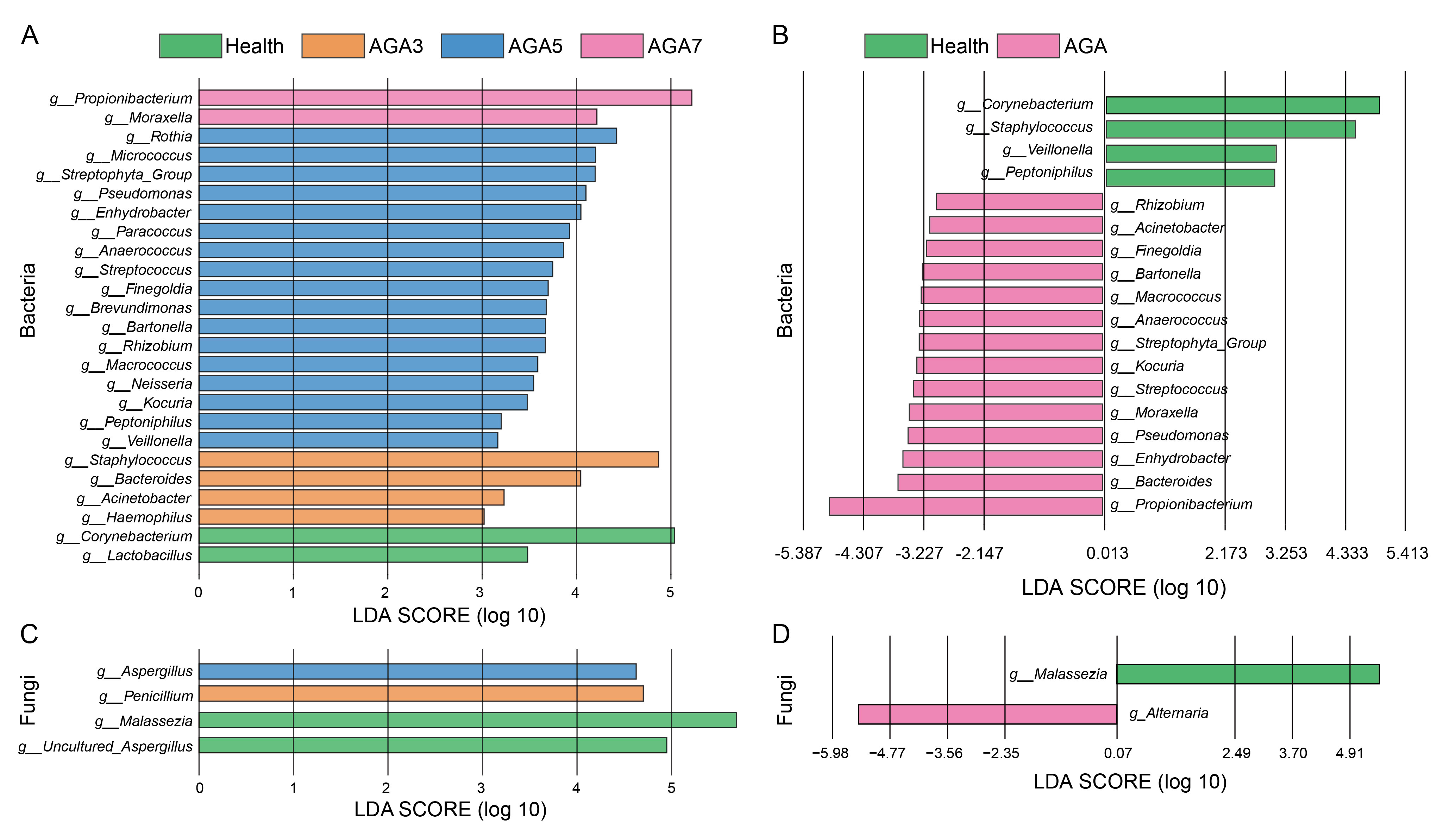


**Fig. S5. LEfSe analysis was performed using bacterial and fungal relative abundance tables, respectively, with an LDA threshold of 4.0.** (A) LDA score histogram of bacterial differential‐abundance features across AGA severity groups. (B) LDA score histogram of bacterial differential‐abundance features across subject groups. (C) LDA score histogram of fungal differential‐abundance features across AGA severity groups. (D) LDA score histogram of fungal differential‐abundance features across subject groups.

**Table S1. Information table of the 89 subjects in the experimental cohort**

| **Sample ID** | **Sex** | **Age** | **AGA severity** | **Age group** |
| --- | --- | --- | --- | --- |
| A34 | male | 49 | Health | age6 |
| A35 | male | 37 | Health | age4 |
| A36 | male | 40 | Health | age5 |
| A37 | male | 43 | Health | age5 |
| A38 | male | 49 | Health | age6 |
| A39 | male | 54 | Health | age7 |
| A40 | male | 53 | Health | age7 |
| A41 | male | 40 | Health | age5 |
| A42 | male | 50 | Health | age7 |
| A43 | male | 46 | Health | age6 |
| A44 | male | 39 | Health | age4 |
| A45 | male | 48 | Health | age6 |
| A46 | male | 58 | Health | age8 |
| A47 | male | 60 | Health | age9 |
| A48 | male | 54 | Health | age7 |
| A49 | male | 44 | Health | age5 |
| A50 | male | 50 | Health | age7 |
| A51 | male | 55 | Health | age8 |
| A52 | male | 63 | Health | age9 |
| A53 | male | 39 | Health | age4 |
| A54 | male | 42 | Health | age5 |
| A55 | male | 44 | Health | age5 |
| A56 | male | 50 | Health | age7 |
| A57 | male | 62 | Health | age9 |
| B13 | male | 55 | AGA3 | age8 |
| B14 | male | 59 | AGA3 | age8 |
| B15 | male | 59 | AGA3 | age8 |
| B16 | male | 54 | AGA3 | age7 |
| B17 | male | 48 | AGA3 | age6 |
| B18 | male | 53 | AGA3 | age7 |
| C06 | male | 35 | AGA5 | age4 |
| C07 | male | 62 | AGA5 | age9 |
| C08 | male | 51 | AGA5 | age7 |
| C09 | male | 45 | AGA5 | age6 |
| C10 | male | 51 | AGA5 | age7 |
| D07 | male | 58 | AGA7 | age8 |
| D08 | male | 43 | AGA7 | age5 |
| D09 | male | 40 | AGA7 | age5 |
| D10 | male | 50 | AGA7 | age7 |
| A01 | male | 25 | Health | age2 |
| A02 | male | 35 | Health | age4 |
| A03 | male | 40 | Health | age5 |
| A04 | male | 32 | Health | age3 |
| A05 | male | 32 | Health | age3 |
| A06 | male | 35 | Health | age4 |
| A07 | male | 24 | Health | age1 |
| A13 | male | 40 | Health | age5 |
| A14 | male | 39 | Health | age4 |
| A15 | male | 32 | Health | age3 |
| A16 | male | 24 | Health | age1 |
| A18 | male | 24 | Health | age1 |
| A19 | male | 27 | Health | age2 |
| A20 | male | 25 | Health | age2 |
| A21 | male | 36 | Health | age4 |
| A22 | male | 24 | Health | age1 |
| A23 | male | 27 | Health | age2 |
| A24 | male | 24 | Health | age1 |
| A25 | male | 25 | Health | age2 |
| A26 | male | 32 | Health | age3 |
| A27 | male | 25 | Health | age2 |
| A28 | male | 25 | Health | age2 |
| A29 | male | 24 | Health | age1 |
| A30 | male | 24 | Health | age1 |
| A31 | male | 24 | Health | age1 |
| A32 | male | 26 | Health | age2 |
| A33 | male | 24 | Health | age1 |
| B01 | male | 25 | AGA3 | age2 |
| B02 | male | 30 | AGA3 | age3 |
| B03 | male | 36 | AGA3 | age4 |
| B04 | male | 39 | AGA3 | age4 |
| B05 | male | 26 | AGA3 | age2 |
| B06 | male | 24 | AGA3 | age1 |
| B07 | male | 42 | AGA3 | age5 |
| B08 | male | 20 | AGA3 | age1 |
| B09 | male | 44 | AGA3 | age5 |
| B10 | male | 24 | AGA3 | age1 |
| B11 | male | 28 | AGA3 | age2 |
| B12 | male | 25 | AGA3 | age2 |
| C01 | male | 46 | AGA5 | age6 |
| C02 | male | 42 | AGA5 | age5 |
| C03 | male | 34 | AGA5 | age3 |
| C04 | male | 45 | AGA5 | age6 |
| C05 | male | 24 | AGA5 | age1 |
| D01 | male | 54 | AGA7 | age7 |
| D02 | male | 40 | AGA7 | age5 |
| D03 | male | 36 | AGA7 | age4 |
| D04 | male | 38 | AGA7 | age4 |
| D05 | male | 47 | AGA7 | age6 |
| D06 | male | 60 | AGA7 | age9 |

**Table S2. ANOVA of Shannon diversity by meta variables.** (A) and (B) show the statistical significance (*p-value*) quantifying the relative contribution of various factors to the alpha diversity of scalp bacterial and fungal microbiota, where * indicates *p-value* < 0.05, ** indicates *p-value* < 0.01, and NS denotes not significant.

**A. Analysis of variance of meta variables on bacteria Shannon diversity.**

| **Variable** | **Kingdom** | **Sum_square** | ***F*** | ***p-value*** |
| --- | --- | --- | --- | --- |
| Position | bacteria | 0.40 | 3.90 | * |
| Age | bacteria | 1.32 | 1.62 | NS |
| AGA severity | bacteria | 20.99 | 68.71 | ** |
| Subjects | bacteria | 0.93 | 9.12 | ** |

**B. Analysis of variance of meta variables on fungi Shannon diversity.**

| **Variable** | **Kingdom** | **Sum_square** | ***F*** | ***p-value*** |
| --- | --- | --- | --- | --- |
| Position | fungi | 0.02 | 0.26 | NS |
| Age | fungi | 0.32 | 0.51 | NS |
| AGA severity | fungi | 1.60 | 6.81 | ** |
| Subjects | fungi | 0.01 | 0.07 | NS |

**Table S3. Spearman correlation of selected age-related taxa with actual age.** The Spearman method was used to test the correlation between sample age and target genus, where * indicates *p-value* < 0.05, ** indicates *p-value* < 0.01, and NS denotes not significant.

| **Genus** | **Spearman correlation coefficient** | ***p-value*** |
| --- | --- | --- |
| *g__Paracoccus* | 0.67 | ** |
| *g__Micrococcus* | 0.57 | ** |
| *g__Rothia* | 0.53 | ** |
| *g__Propionibacterium* | -0.47 | ** |
| *g__Acinetobacter* | -0.49 | ** |
| *g__Cladosporium* | 0.55 | ** |

**Table S4. Multiple hypothesis testing was controlled using the False Discovery Rate (FDR) correction method, and for each taxon, the original *p-value* (Kruskal-Wallis), FDR-adjusted *q-value*.** Nonparametric difference tests were performed for bacterial (A) and fungal (B) microbial abundance data according to AGA severity groups, and all *p-value* were corrected by FDR, where * indicates *p-value* < 0.05, ** indicates *p-value* < 0.01, and NS indicates not significant.

**A. FDR *q‑value* and *p‑value* for bacteria taxa across AGA severity groups.**

| **Sample ID** | **Kingdom** | **FDR *q-value*** | ***p-value*** |
| --- | --- | --- | --- |
| *g__Macrococcus* | Bacteria | 6.28E-15 | ** |
| *g__Kocuria* | Bacteria | 6.28E-15 | ** |
| *g__Peptoniphilus* | Bacteria | 2.37E-09 | ** |
| *g__Bacteroides* | Bacteria | 2.37E-09 | ** |
| *g__Acinetobacter* | Bacteria | 3.43E-09 | ** |
| *g__Enhydrobacter* | Bacteria | 1.29E-08 | ** |
| *g__Streptococcus* | Bacteria | 1.82E-08 | ** |
| *g__Finegoldia* | Bacteria | 2.20E-08 | ** |
| *g__Moraxella* | Bacteria | 5.52E-08 | ** |
| *g__Propionibacterium* | Bacteria | 1.97E-07 | ** |
| *g__Pseudomonas* | Bacteria | 2.35E-07 | ** |
| *g__Corynebacterium* | Bacteria | 2.35E-07 | ** |
| *g__Veillonella* | Bacteria | 1.43E-06 | ** |
| *g__Paracoccus* | Bacteria | 1.43E-06 | ** |
| *g__Rhizobium* | Bacteria | 3.99E-06 | ** |
| *g__Micrococcus* | Bacteria | 7.60E-06 | ** |
| *g__Brevundimonas* | Bacteria | 9.76E-06 | ** |
| *g__Staphylococcus* | Bacteria | 2.94E-05 | ** |
| *g__Anaerococcus* | Bacteria | 4.94E-05 | ** |
| *g__Bartonella* | Bacteria | 1.07E-04 | ** |
| *g__Streptophyta_Group* | Bacteria | 1.54E-04 | ** |
| *g__Rothia* | Bacteria | 3.38E-04 | ** |
| *g__Haemophilus* | Bacteria | 3.33E-03 | ** |
| *g__Lactobacillus* | Bacteria | 3.37E-03 | ** |
| *g__Neisseria* | Bacteria | 6.21E-03 | NS |

**B. FDR *q‑value* and *p‑value* fungi taxa across AGA severity groups**

| **Sample ID** | **Kingdom** | **FDR *q-value*** | ***p-value*** |
| --- | --- | --- | --- |
| *g__Malassezia* | Fungi | 1.04E-15 | ** |
| *g__Alternaria* | Fungi | 5.17E-13 | ** |
| *g__Penicillium* | Fungi | 2.90E-07 | ** |
| *g__Aspergillus* | Fungi | 1.22E-06 | ** |
| *g__Uncultured_Aspergillus* | Fungi | 6.24E-03 | ** |

**Table S5. The set of parameter combinations exhaustively searched by GridSearchCV**

| **Model parameters** | **Parameter ranges** |
| --- | --- |
| n_estimators | 200, 225, 250, 275, 300, 325, 350, 375, 400 |
| min_samples_split | 2, 3 |
| min_samples_leaf | 2, 3, 4 |
| max_features | None, log2, sqrt |
| max_depth | 8, 10, 12, 15 |
